# Testicular metastasis as the first clinical manifestation of pancreatic adenocarcinoma: a case report

**DOI:** 10.1186/s13256-015-0626-4

**Published:** 2015-06-12

**Authors:** Luigi Cormio, Francesca Sanguedolce, Paolo Massenio, Giuseppe Di Fino, Mariantonietta Bruno, Giuseppe Carrieri

**Affiliations:** 1 Department of Urology and Renal Transplantation, University of Foggia, Foggia, Italy; 2 Department of Pathology, University of Foggia, Foggia, Italy; 3 Department of Pathology, Madonna delle Grazie Hospital, Matera, Italy

**Keywords:** Immunohistochemistry, Pancreatic cancer, Testicular metastasis

## Abstract

**Introduction:**

Testicular metastases from pancreatic carcinomas are extremely rare and are usually seen in the late phase of the disease. Fewer than 10 cases have been reported in the literature to date, all of which occurred in patients more than 50 years old. Herein we describe the first case, to our knowledge, of testicular metastasis as the first clinical manifestation of pancreatic carcinoma in a young adult.

**Case presentation:**

A 36-year-old Caucasian man presented to our institution with an acutely developed severe pain in his right testis. His clinical examination and scrotal ultrasounds were consistent with a tumor involving the entire right testis. The patient underwent radical orchiectomy. His pathologic examination revealed the tumor to be a metastasis from a pancreatic cancer that was confirmed by an abdominal computed tomographic scan.

**Conclusions:**

The present case shows that testicular metastasis could be the presenting sign of metastatic pancreatic cancer and that testicular metastases from solid organ tumors, though typical of elderly people, may occasionally be seen in young adults as well.

## Introduction

Testicular metastases from solid non-lymphoid tumors are extremely rare, accounting for only 0.02% to 3.6% of all testicular neoplastic lesions [1–3]. They usually affect males over 50 to 60 years of age, and it is extremely uncommon for them to present as the first clinical manifestation of the underlying malignancy [4, 5]. Primary sites include, in a roughly descending order, the prostate, lung, skin (melanoma), kidney, digestive tract, and adrenal gland (neuroblastoma). Testicular metastases from the bladder, soft tissue, and penis have been described occasionally [6–8].

In a review of the literature, we found fewer than 10 cases of testicular metastases arising from a pancreatic neoplasm, all of which occurred in patients more than 50 years old [4–7]. Herein we describe the first case, to our knowledge, of testicular metastasis as the first clinical manifestation of pancreatic carcinoma in a young adult.

## Case presentation

**Fig. 1:**
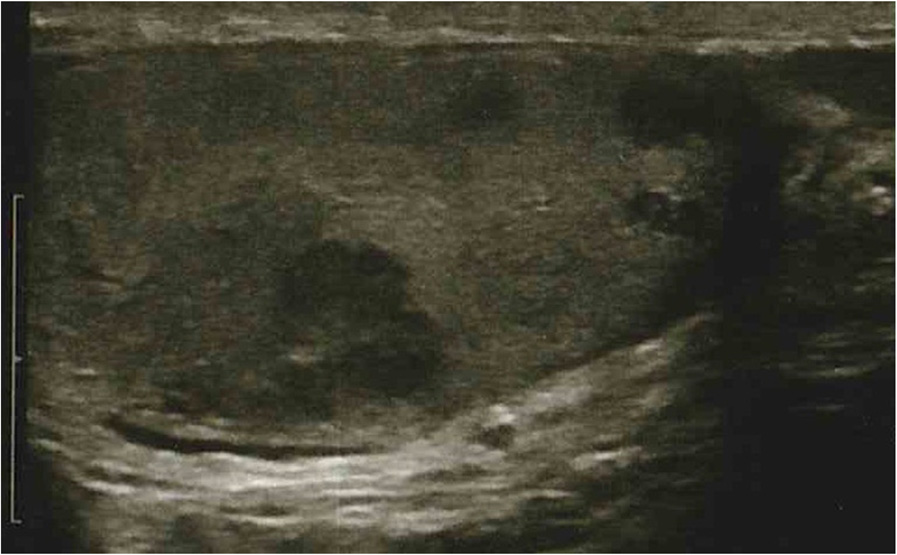
Scrotal ultrasound. Structural changes of the entire right testis

**Fig. 2:**
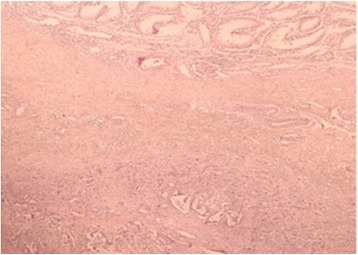
Histologic examination revealed a metastatic adenocarcinoma to the testis with residual seminiferous tubules (*top*). Hematoxylin and eosin stain; original magnification, 400×

A 36-year-old Caucasian man presented to our institution with an acutely developed severe pain in his right testis. One year before this presentation, owing to azoospermia, he had undergone bilateral testicular biopsy and sperm retrieval that led to pregnancy. Clinical examination disclosed bilateral hypogonadism with a hard and nodular right testis and absence of palpable inguinal and pelvic nodes. Scrotal ultrasounds showed marked structural changes of the entire right testis (Fig. 1). A pre-operative chest X-ray and blood tests, including the β-subunit of human chorionic gonadotropin (β-hCG) and α-fetoprotein (AFP), were all normal. The patient underwent right radical orchiectomy. His pathologic examination revealed infiltration of the testis by a poorly differentiated carcinoma with mucinous and micropapillary features (Fig. 2). The cancer-free testicular tissue showed mostly regular tubules with normal spermatogenesis, despite the macroscopic findings. Careful examination of the entire lesion allowed us to rule out teratoma. Immunohistochemically, the tumor was positive for cytokeratin (CK) 7 and CK20 (focal) and negative for placental alkaline phosphatase (PLAP), AFP, β-hCG, calretinin, and inhibin, with a Ki67/MIB1 proliferation index of 15%.

**Fig. 3:**
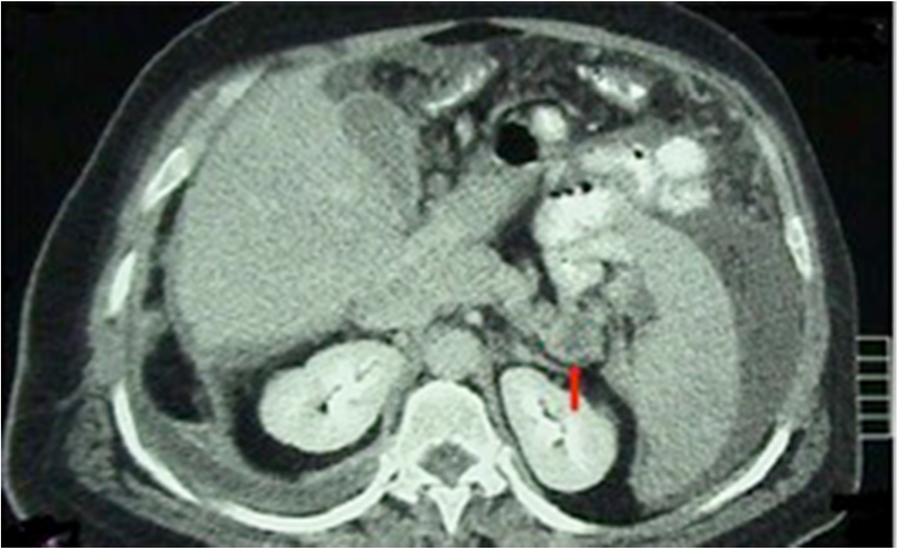
Computed tomographic scan. Densitometric alteration of the tail of the pancreas can be seen (inhomogeneous hypodensity, red arrow)

Taken together, such findings allowed us to rule out a primitive testicular cancer as well as metastasis from hepatocellular carcinoma, and they were consistent with metastatic adenocarcinoma of the pancreas. Chest and abdominal computed tomography demonstrated the presence of a tumor of the tail of the pancreas (Fig. 3), along with hepatic metastases. There was no evidence of retroperitoneal lymphadenopathy. Serum tumor markers such as cancer antigen (CA) 125 and CA 19-9 levels were within normal range. The patient was given chemotherapy but died 3 months later.

## Discussion

The low incidence of secondary tumors to the testis is apparently due to the lower temperature of the scrotum, which could impair the metastatic ability of tumor cells from solid organs [7, 9].

Various routes of spread of metastatic tumors to the testes have been suggested, including arterial embolization, retrograde venous spread, retrograde lymphatic spread, direct spread along the vas deferens to the epididymis, and transperitoneal seeding through a patent tunica vaginalis. The dissemination mechanism seems to vary with type and location and the primary tumor, and one does not exclude the other [3, 10]. In general, for primary tumors that are located outside the pelvic and abdominal cavity, arterial dissemination seems to be the preferred route [11]. Given the absence of retroperitoneal lymphadenopathy, this was likely the case in our patient. Transperitoneal seeding is probable when peritoneal carcinomatosis is present [12].

Testicular metastases usually are a final manifestation of widely disseminated tumors, and therefore they often go unnoticed [6]. Rarely, they represent the first clinical manifestation of the underlying malignancy [4, 5], thus simulating a primary testicular tumor, as in our patient.

Clinically, there are no specific features that differentiate primary from secondary testicular tumors, as both may present with local pain, tenderness, nodular lesions, or an indurated testis; painless swelling, however, points toward the diagnosis of a testicular tumor. Also, serum tumor markers such as AFP and β-hCG are not helpful in distinguishing primary from secondary testicular tumors. On the one hand, normal values may be consistent with primary as well as secondary malignancies; on the other hand, elevated values may be related to gonadal as well as extragonadal tumors [5]. Interestingly, one of the few reported cases of pancreatic adenocarcinoma presenting as a testicular tumor featured raised hCG levels due to extragonadal secretion [13]. However, that patient was 77 years old, thus suggesting the testicular tumor to be a secondary rather than a primary malignancy. As a matter of fact, the patient’s age may play an important role in differentiating a primary tumor from a secondary one. Patients with testicular metastases are older than those with primary testicular tumors [14–16], as the peak incidence of testicular metastases from all tumors is in the sixth decade of life, according to several large series reported in literature. Therefore, the peculiarity of our patient is that he was a young adult with bilateral hypogonadism and azoospermia, features that pointed toward the diagnosis of a primary testicular tumor. Pathologic examination is essential to differentiating primary from secondary testicular tumors. The first important feature is the absence of intratubular malignant germ cell neoplasms [16]. In the large series reported by García-González *et al*. [6], two architectural patterns were identified: an interstitial one, which can also be seen in primary tumors, though less frequently, and a destructive one, which was present in our patient. According to Price and Mostofi [14], another feature that increases the possibility of metastatic rather than primary tumor is the presence of tumor emboli in the vascular channels of the parenchyma and tunica albuginea, though intravascular invasion was very rarely found in another small series [6]. In our patient, no lymphovascular invasion was detectable in the testis.

Immunohistochemistry is nowadays regarded as the most sensitive and specific way of determining the origin of the tumor. Recently, new markers have been developed and are being used in diagnosis. These include homeobox protein NANOG, SOX2, and Oct-3/4 [17, 18]. In our patient, the histological diagnosis relied on positive immunohistochemical staining for CK7, CK20 (focal), and negative staining for PLAP, AFP, β-hCG, calretinin, inhibin, and a Ki67/MIB1 proliferation index of 15%, findings that were altogether consistent with metastatic adenocarcinoma of the pancreas. Serum CA 125 and CA 19-9 levels were within normal range, but abdominal computed tomography revealed the pancreatic tumor along with the hepatic metastases.

## Conclusions

The present case shows that testicular metastasis can be the presenting sign of metastatic pancreatic cancer and that testicular metastases from solid organ tumors, though typical of elderly people, may occasionally be seen in young adults as well.

## Consent

Written informed consent was obtained from the patient’s next of kin for publication of this case report and any accompanying images. A copy of the written consent is available for review by the Editor-in-Chief of this journal.

## Abbreviations

AFP: α-Fetoprotein
β-hCG: β-Subunit of human chorionic gonadotropin
CA: Cancer antigen
CK: Cytokeratin
PLAP: Placental alkaline phosphatase
